# Intravenous Amoxicillin Plus Intravenous Gentamicin for Children with Severe Pneumonia in Bangladesh: An Open-Label, Randomized, Non-Inferiority Controlled Trial

**DOI:** 10.3390/life11121299

**Published:** 2021-11-26

**Authors:** Lubaba Shahrin, Mohammod Jobayer Chisti, Monira Sarmin, Abu Sayem Mirza Md. Hasibur Rahman, Abu Sadat Mohammad Sayeem Bin Shahid, Md. Zahidul Islam, Farzana Afroze, Sayeeda Huq, Tahmeed Ahmed

**Affiliations:** 1 Head Acute Respiratory Infection Unit, Dhaka Hospital, Nutrition and Clinical Services Division, International Centre for Diarrhoeal Disease Research, Bangladesh (icddr,b), Dhaka 1000, Bangladesh; 2 Head Clinical Research Unit, Nutrition and Clinical Services Division, International Centre for Diarrhoeal Disease Research, Bangladesh (icddr,b), Dhaka 1000, Bangladesh; chisti@icddrb.org; 3 Nutrition and Clinical Services Division, International Centre for Diarrhoeal Disease Research, Bangladesh (icddr,b), Dhaka 1000, Bangladesh; drmonira@icddrb.org (M.S.); sayem@icddrb.org (A.S.M.M.H.R.); sayeem@icddrb.org (A.S.M.S.B.S.); zahid.islam@icddrb.org (M.Z.I.); farzanaafroz@icddrb.org (F.A.); sayeeda@icddrb.org (S.H.); 4 International Centre for Diarrhoeal Disease Research, Bangladesh (icddr,b), Dhaka 1000, Bangladesh; tahmeed@icddrb.org

**Keywords:** amoxicillin, severe pneumonia, randomized controlled trial, children treatment failure

## Abstract

The World Health Organization (WHO) recommends intravenous (IV) ampicillin and gentamicin as first-line therapy to treat severe pneumonia in children under five years of age. Ampicillin needs to be administered at a six-hourly interval, which requires frequent nursing intervention and bed occupancy for 5–7 days, limiting its utility in resource-poor settings. We compared the efficacy of IV amoxicillin over IV ampicillin, which is a potential alternative drug in treating severe pneumonia in children between 2–59 months. We conducted an unblinded, randomized, controlled, non-inferiority trial in the Dhaka hospital of icddr,b from 1 January 2018 to 31 October 2019. Children from 2–59 months of age presenting with WHO defined severe pneumonia with respiratory danger signs were randomly assigned 1:1 to either 50 mg/kg ampicillin or 40 mg/kg amoxicillin per day with 7.5 mg/kg gentamicin. The primary outcome was treatment failure as per the standard definition of persistence of danger sign(s) of severe pneumonia beyond 48 h or deterioration within 24 h of therapy initiation. The secondary outcomes were: (i) time required for resolution of danger signs since enrolment, (ii) length of hospital stay, (iii) death during hospitalization, and (iv) rate of nosocomial infections. Among 308 enrolled participants, baseline characteristics were similar among the two groups. Sixty-two (20%) children ended up with treatment failure, 21 (14%) in amoxicillin, and 41 (27%) in ampicillin arm, which is statistically significant (relative risk [RR] 0.51, 95% CI 0.32–0.82; *p* = 0.004). We reported 14 deaths for serious adverse events, 4 (3%) and 10 (6%) among amoxicillin and ampicillin arm, respectively. IV amoxicillin and IV gentamicin combination is not inferior to combined IV ampicillin and IV gentamicin in treating severe pneumonia in under-five children in Bangladesh. Considering the less frequent dosing and more compliance, IV amoxicillin is a better choice for treating children with severe pneumonia in resource-limited settings.

## 1. Introduction

Etiology analysis of community-acquired severe pneumonia in children under five years of age estimated that although viruses are predominant in this age group, those required hospital admission are mostly caused by bacteria [1]. Streptococcus pneumoniae is most prevalent in this age group and constituted 40% of isolation cases [1]. Children hospitalized with the World Health Organization (WHO) defined severe pneumonia are recommended to treat with intravenous (IV) ampicillin plus IV gentamicin combination [2]. Ampicillin is a penicillin derivative, requires daily four times parenteral administration for 5 to 7 days [3,4]. This antibiotic treatment not only requires frequent nursing engagement but also incurs hospitalization costs and undue attendance of the family [5]. Many resource-limited settings, including Bangladesh, where the nurse-patient ratio is high, bed occupancy is limited, nosocomial infection is challenging to tackle, exploring alternative antibiotics is a great demand [5,6]. 

In the search for alternative antibiotics, clinical trials have already identified amoxicillin as being equally efficacious compared to ampicillin in treating severe bacterial infection in young infants [7,8,9]. However, no head-to-head trial was done in children between 2 and 59 months of age presenting with severe pneumonia. We opted for amoxicillin because structurally it is closely related to ampicillin and similar range in the spectrum of activity and potency. Following a dose, amoxicillin achieves two times higher concentration in blood than ampicillin and is equally effective in oral form; it can be administered 12-hourly daily [10]. UNICEF has already listed this drug as “priority essential” and recommended the oral formulation to treat non-severe pneumonia in under-five children [11,12]. 

As antibiotic choice and recommendation are not constant over time and depend on socio-demographic factors of low- and middle-income countries, a simple regimen with fewer injections is essential. In our setting, the primary reason for choosing IV amoxicillin over ampicillin is the cost, which is four times less as per market value in the study site, Bangladesh. Moreover, due to fewer IV interventions, we assume parental satisfaction can be high, and readiness to complete the treatment can be more as indicated by a previous study [13]. Chances of nosocomial infection will be low due to less contact with the health-care provider. IV amoxicillin can be switched to oral amoxicillin after 72 h [3,9], if a child has early improvement or expectant recovery. Should our trial be successful, amoxicillin can be implemented in similar settings based on the affordable cost, compliance, parent’s satisfaction, and ease of switching to ambulatory service. 

To date, no clinical trial has assessed the efficacy of twice-daily amoxicillin versus four-times daily ampicillin in children from 2–59 months of age presenting with severe pneumonia, and thus we aimed to achieve this goal. The objective of this study was to compare the efficacy of a potential alternative drug, amoxicillin, which requires 12 hourly IV administrations and thus less-frequent nursing intervention, and can be switched to oral form after clinical improvement. 

The primary hypothesis for this trial was that IV amoxicillin would not be inferior to IV ampicillin for treating severe pneumonia in a resource-limited setting. Our secondary hypothesis was that IV amoxicillin group would not be inferior to IV ampicillin across broader assessments of resolution time, hospital stay, nosocomial infection and death.

## 2. Materials and Methods

### 2.1. Study Design and Patients

We conducted an open-label, randomized, non-inferiority, controlled trial with the parallel design with a 1:1 treatment allocation. The study received approval from the Institutional Review Board of International Centre for Diarrheal Disease Research, Bangladesh (icddr,b) (approval No. PR-17061, version 4.0, dated 4 July 2018). The trial was registered at the NIH clinical trial registry on 11 December 2017, Reg number: NCT 03369093. Before enrollment into the study, written informed consent was obtained from the legal guardians of the participants. Participation was voluntary, and non-participation did not hamper the standard hospital management. All the methods above were carried out in accordance with relevant guidelines and regulations (Declarations of Helsinki).

### 2.2. Study Settings

Participants were recruited in the Dhaka Hospital of icddr,b, from 1 January 2018 to 31 October 2019. This is the largest diarrheal disease hospital in the world, where 166,000 patients, mostly from poor socio-economic backgrounds, sought treatment in the year 2019. The patients usually presented with diarrhea, pneumonia, malnutrition, sepsis, and other associated complications and received free of cost treatment from the hospital. Details of the other facility of the settings are described elsewhere [14]. A data safety and monitoring board (DSMB) worked independently to monitor serious adverse events (SAE)s related to treatment.

### 2.3. Inclusion Criteria

Eligibility criteria were children aged 2–59 months, with severe pneumonia as defined by the WHO [3]: based on cough and difficulty in breathing plus at least two of the followings: (i) central cyanosis or oxygen saturation < 90% on pulse oximetry, (ii) severe respiratory distress (e.g., grunting, very severe chest indrawing), (iii) signs of pneumonia with a general danger sign (e.g., inability to breast-fed or drink, lethargy or unconscious, convulsion). 

### 2.4. Exclusion Criteria

Exclusion criteria against recruitment were children with known antibiotic therapy at home or other hospital for ≥48 h, known congenital or chromosomal anomaly (e.g., congenital heart disease, laryngomalacia, cleft lip, cleft palate, and trisomy 21). Also, we did not recruit children who presented with gasping respiration or required cardio-pulmonary resuscitation or referral to the higher treatment facility for critical serum creatinine value.

### 2.5. Randomization and Masking

Upon obtaining the informed consent, recruited children were randomized in equal numbers into two groups. The random assignment was performed with a block number of 4 by computer-generated urn randomization algorithm [15] numbers were used with a prefixed block number unknown to the researcher. The randomization sequence was prepared before the commencement of the study by an independent statistician at icddr,b. For masking the treatment groups, randomization numbers were provided to the principal investigator (L.S) in sequentially numbered, sealed, opaque envelopes containing the name of the treatment on a card inside the envelope. The study physician (ASMMHR) opened the next numbered envelope in the presence of another non-study physician of the designated treatment area. One group of participants was allocated for the WHO standard treatment with IV ampicillin and IV gentamicin, and the other group for IV amoxicillin and IV gentamicin. Study medicines were donated by Square Pharmaceuticals Ltd., Dhaka, Bangladesh. As it was an open level trial, masking of the injection vials was not suggested by the DSMB.

### 2.6. Procedures

All the children received a thorough clinical assessment at the hospital triage, and if required immediate care and resuscitation were given at the outset according to standard hospital protocol. This includes but is not limited to the correction of dehydration, oxygen supplementation (if SpO_2_ < 90%), correction of hypoglycemia and electrolyte imbalance, and initial management of severe acute malnutrition [16,17]. At enrollment, baseline data, demographic and social information, detailed clinical examination, and vital signs, including pulse oximetry and anthropometric measurements, were collected using standardized case report form. Oxygen saturation was measured using a pulse oximeter (Nellcor Puritan Bennett Inc. N-560, Made in Seoul, Korea) with a probe on a finger or toe when the child breathes in room air. Relevant information, including medical history, socio-demographic characteristics, feeding history, immunization status, history of contact for tuberculosis, recent and past respiratory tract infection of patient and any family members was recorded. Critically ill patients were managed as per the standard hospital practice [18,19,20,21]. 

Based on standard operating procedure, clinical improvement was expected within 72 h of standard treatment. An improvement was defined by the absence of danger signs, resolution of hypoxemia, adequate oral feeding, and improvement of wellbeing and activity. Once clinical improvement was declared, injection ampicillin or injection amoxicillin were switched to oral amoxicillin 40 mg/kg/dose 12 h with ongoing injection gentamicin. Children having early improvement with resolution of all danger signs were advised for discharge and requested to visit ambulatory medical support where they would receive remaining doses of intramuscular gentamicin.

Laboratory measurements: As per the standard treatment protocol of the hospital, complete blood count (CBC), and chest X-ray were done for children who also had severe acute malnutrition. Serum electrolyte and creatinine were performed for suspected electrolyte imbalance, and blood culture for presumed sepsis. Operating laboratory devices used for the above tests were for CBC (XN 1000, Sysmex, Kobe, Japan), serum electrolytes (AU680, Beckman Coulter, Brea, CA, USA), and blood culture (BacTAlert 3D, bioMérieux, Lyon, France). All the tests were carried out in the ISO accredited Laboratory Sciences and Services Division of, icddr,b in the study premises.

Standard treatment group: The WHO standard treatment group, participants received an IV ampicillin 50 mg/kg/dose 6 hourly and an IV gentamicin 7.5 mg/kg once daily for 5–7 days. 

Intervention group: On the other hand, in the intervention group (amoxicillin group) the patient received IV amoxicillin 40 mg/kg/dose 12 hourly and IV gentamicin 7.5 mg/kg once daily for 5–7 days. Children in both regimens required IV cannula in place for the administration of study medications. 

### 2.7. Primary Outcome Measures

The primary outcome of this trial was treatment failure [7,18], defined as either persistence of danger signs (central cyanosis, hypoxemia, grunting, inability to breast-fed or drink, lethargy or unconscious or convulsion) at the end of 48 h or appearance of new danger signs (requiring mechanical ventilation, developing severe sepsis or septic shock, death or referral to another specialized hospital for acute kidney injury or paralytic ileus) within 24 h of enrolment. In the intervention trial of drugs or devices, efficacy is usually determined by assessing treatment failure among the treatment groups, which we had followed in our trial as per the opinion of the institutional scientific committee [6,8,16,19,20].

### 2.8. Secondary Outcome Measures

Predefined secondary outcomes included: (i) time to resolution of danger signs of severe pneumonia; (ii) length of hospital stay; (iii) rate of nosocomial infection; (iv) death.

### 2.9. Discontinuation Policy

There were some criteria for discontinuing the allocated interventions upon fulfilling treatment failure criterion:

1. No improvement of danger signs after 48 h of starting antibiotic therapy
2. Clinical deterioration of the patient in terms of hypoxemia, grunting, or respiratory failure requiring mechanical ventilation
3. Development of septic shock or severe sepsis
4. Required a referral to another hospital for conditions such as acute kidney injury (raised serum creatinine) during treatment period

In such instances, ongoing medications were discontinued, and the patient was switched to second-line antibiotics (IV ceftriaxone 100 mg/kg once daily plus IV levofloxacin 10 mg/kg once daily) and continued for 5–7 days based on clinical improvement [21]. For further evaluation of the status, CBC, blood culture, chest x-ray, and serum electrolytes tests were performed as per hospital policy. For the confirmed cases of acute kidney injury [22], participants were referred to a specialized hospital with a pediatric nephrology unit.

### 2.10. Data Analytic Strategy

Data were analyzed from 01 November 2019 to 30 June 2020. All the children randomized to treatment groups were included in the analysis regardless of the number of treatment failure or study outcome. The sample size calculation assumed that enrollment of 154 participants in each group (total = 308) had 80% power and significance level (α) of 0.025, details was published elsewhere [18]. Power calculations were done following the formula of Julious to guarantee a power of 80% for assessing noninferiority while accommodating the repeated-measures design [23]. According to protocol, intention to treat analysis was done to understand the statistical difference of primary outcome (treatment failure) between intervention and control group.

We analyzed data performed by the SPSS (Statistical Package for the Social Sciences- version 20.0 Windows) (SPSS, Chicago, IL, USA), STATA (Statistics and DATA: version 13), and Epi Info (version 7.0, USD, Stone Mountain, GA, USA). We calculated relative risk (RR) and their 95% CIs to express the primary outcome. We used Mann–Whitney test for the continuous variable; for length of hospital stay, we performed survival analysis by Kaplan–Meier plot and also calculated the hazard ratio (HR) by Cox’s proportional hazard model. We adjusted the variables in a multivariable model those were significant at a 5% level of significance at bi-variate analysis.

## 3. Results

### 3.1. Participants Distribution by Subgroup

**Figure 1:**
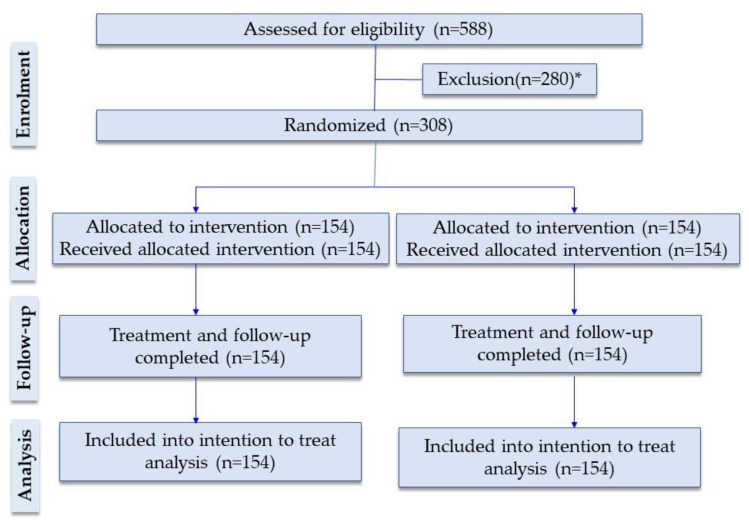
Amoxicillin trial profile showing participant enrollment. * The causes of exclusion are given in Supplementary Table S1.

We screened 588 children aged 2–59 months with severe pneumonia and respiratory danger signs for eligibility between 1st January 2018 and 31st October 2019. Among them, 308 children (52% of total screened) fulfilled the study inclusion criteria (Figure 1) and were randomly assigned to either amoxicillin group (154) or ampicillin group (154). The rest (274) were excluded as 193 (70%) had proof of receiving antibiotics, 60 (21%) had suspected or confirmed congenital or chromosomal anomaly or cerebral palsy; 20 (7.3%) had serious illness and candidate of assisted ventilation and the guardian of 1 (one) patient refused to participate in the study (Supplementary Table S1).

Except total carbon di-oxide (TCO_2_), the distribution of baseline characteristics was comparable between the groups shown in Table 1 and Table 2. Among all the participants’ male child was predominant (62%) and the mean age was 9.43 months. Based on nutritional status, 25% participants had severe stunting in amoxicillin group and 12% participants had nutritional edema in ampicillin group, however it was not statistically significant. Enrollment criteria were also comparable among the groups (Table 2).

The mean duration of receiving amoxicillin and ampicillin was (48.5) hours and (53.0) hours, respectively. For the treatment of hypoxemia 48 (16%) participants received bCPAP, of them 25 (16%) and 23 (15%) children received IV amoxicillin and IV ampicillin, respectively. Besides them, participants who developed hypoxemia after admission were categorized as treatment failure. 73 (24%) of the participants had severe acute malnutrition during enrolment, after recovering from the acute phase, 18 (25%) were convinced in continuing the treatment in nutrition rehabilitation unit, of which 9 (50%) and 9 (50%) belonged to amoxicillin and ampicillin group, respectively.

### 3.2. Treatment Failure by Groups

Sixty-two (20%) children ended up with treatment failure, 21 (14%) in amoxicillin, and 41 (27%) in ampicillin group (Table 3). Children who received IV amoxicillin had a 49% lower risk of treatment failure compared to IV ampicillin, which was statistically significant (95% CI 0.32, 0.82; *p*-value 0.004). In amoxicillin group, 21 (14%) children experienced treatment failure diagnosed by non-resolving/persistence of danger signs 10 (6%) after 48 h and deterioration of clinical condition (i.e., developing septic shock) or appearance of danger signs 11 (7%) within 1st 24 h.

Conversely, among the 41 (27%) treatment failure children in ampicillin group, 20 (14%) children experienced unresolved or persistence of danger signs after 48 h, and 21 (14%) developed new danger signs within 1st 24 h. Distribution of unresolved danger signs during treatment failure were hypoxemia 15 (10%) and 22 (15%) is amoxicillin and ampicillin group respectively; abnormal mentation 08 (5%) and 19 (13%), and feeding difficulty 17 (11%) and 29 (19%), respectively, among amoxicillin and ampicillin groups, respectively.

### 3.3. Hospital Outcome by Groups

Among the participants, we discharged 271 (88%), of whom 142 (87%) and 129 (81%) from amoxicillin and ampicillin group, respectively (Table 4). We reported 14 serious adverse events (SAEs) in the form of in-hospital death and the fatality rate was 4.5%; 4 (3%) deaths occurred among the amoxicillin group and 10 (6%) among the ampicillin group. We referred 14 participants to the outside specialized hospital for acute kidney injury 10 (71%), cardiac failure 01 (7%), and intestinal obstruction 01 (7%). We could not collect further laboratory information of the patients from other admitted hospitals, but received their final outcome over phone. Those who referred, 07 (50%) died and 06 (43%) discharged after recovery. We lost tracking of one patient from the specialized hospital. Besides that, a total of 09 patients left against medical advice (LAMA), of whom 03(2%) and 06(4%) each from amoxicillin and ampicillin group, respectively.

### 3.4. Secondary Treatment Outcome by Groups

In the analysis of secondary outcomes (Table 5), time requiring resolution of hypoxemia and general danger sign, duration of hospital stay, and rate of nosocomial infection, were comparable between the groups. 

Survival analysis conducted at day 7 of hospitalization after adjusting for hospital complications, severe sepsis, gender, age in month, and rehydration fluid administered, children in amoxicillin group were 5% more likely to be discharged from the hospital at any time point but this was not statistically significant (Table 6).

## 4. Discussion

To our knowledge, this is the first trial comparing the efficacy of twice-daily IV amoxicillin with four-times daily IV ampicillin in the treatment of severe pneumonia in children between 2–59 months of age. The trial results indicate that IV amoxicillin is not inferior to IV amoxicillin in treating children with severe pneumonia. Eighty-eight percent of our enrolled patients were discharged from hospital after recovery, 3% left against medical advice, 5.4% referred to other medical facility and remaining 5.4% expired. This proportion of hospital outcome is similar to that of other published report from this study site [14]. Our results demonstrated that children who received IV amoxicillin and IV gentamicin experienced 56% less treatment failure than children who received IV ampicillin and IV gentamicin. Although this study was designed and powered to establish non-inferiority, findings showed that our intervention combination was better than the current practice.

A previous study reported treatment failure rate with current ampicillin and gentamicin combination was 58% [21], whereas our study showed 27% treatment failure with the same antibiotic combination. Reason of this big difference may be practicing the locally made innovative bCPAP in treating hypoxemia. This low-cost bCPAP was shown to have significant reduction of mortality among children with severe pneumonia with hypoxemia compared to those who received the WHO standard low flow O_2_ therapy [16]. The trial was accomplished on July 2013. Since then bCPAP was incorporated as part of the standard of care for the children of pneumonia and hypoxemia. Data from the post-trial implementation of bCAP in treating severe pneumonia with hypoxemia (August 2013–December 2017) showed that the mortality and treatment failure is consistent with the trial period and had significant reduction of childhood pneumonia related treatment failure and mortality in the hospital compared to pre-trial period [16] and this may contribute to the overall reduction rate of treatment failure. Overall improvement of pneumonia severity after mass immunization against Hib and pneumococcal infection potentially contributed for the reduction of treatment failure [20,23]. No study used the IV amoxicillin and IV gentamicin combination in treating children with severe pneumonia; therefore, we could not comment on the changing trend of treatment failure rate. Treatment with oral amoxicillin had already been found equally effective in non-severe pneumonia compared to IV ampicillin plus IV gentamicin or IV penicillin [20,24]. Nonetheless, considering Streptococcus pneumoniae was the most common isolates, amoxicillin was equally effective in treating infants with bacterial infection [7,8]. As IV amoxicillin had never been used in severe pneumonia, chances of developing resistance were low; on the contrary, a study from the same settings in Dhaka, Bangladesh, reported that a small number of bacteria isolated from children with severe pneumonia were resistant to IV ampicillin and IV gentamicin [21].

Potential benefits of choosing IV amoxicillin over IV ampicillin are, firstly, that it is equally effective as ampicillin in killing microorganism. Secondly, administering the drug two-times a day requires less nursing intervention and reduced logistical requirements, such as syringes. Besides that, nurses can utilize their time for other patient-related activities. Thirdly, amoxicillin is cheaper than ampicillin; for a five-day course, the cost of IV amoxicillin is only one-fourth of IV ampicillin. Fourthly, after resolution of danger signs IV amoxicillin can be switched to oral form and the child can be discharged for ambulatory care, here once daily gentamicin can be continued in the intramuscular route from ambulatory care or from a local pharmacy. This is acceptable and affordable steps for the family increases drug compliance. Last but not the least; it would reduce the risk of hospital-acquired infection.

This study had several potential limitations. The first limitation was the unblinding of the treatment arms. The second limitation was not using a placebo, however as there is a recommendation of combination of antibiotics, we are not allowed the omission of treatment in such a life-threatening condition. To limit chances of differential misclassification, a second opinion from the non-study ICU consultant was taken for every patient before declaring treatment failure. Another limitation was the single-center study, but due to limited funding we could not include another study site. Finally, we were unable to identify the etiology of pneumonia and their antibiotic resistance pattern in all the cases due to constraint of fund.

Even after these limitations, considering the rate of co-morbidities, such as acute and chronic malnutrition and sepsis, the results of this clinical trial could be applicable to other resource-limited hospitals where severe pneumonia related admission is high with such co-morbidities and the nurse-patient ration is inadequate. 

## 5. Conclusions

The new treatment regimen with IV amoxicillin and IV gentamicin are not less effective than the WHO recommended current therapy with IV ampicillin and IV gentamicin. This new treatment regime may be implemented as the part of the standard of care in resource-limited settings where IV ampicillin and IV gentamicin in combination is still the standard of care. Based on this evidence, Dhaka hospital of icddr,b has recently adopted the use of IV amoxicillin in children suffering severe pneumonia children. 

## Figures and Tables

**Table 1:** Baseline characteristics by treatment arms.

Variables	Overall (n = 308)	Amoxicillin Group (n = 154)	Ampicillin Group (n = 154)
**Demographic and anthropometric characteristics**			
Age in months (median, IQR)	308	7.2 (4.66, 11.14)	7.05 (4.34, 11.04)
Male gender,	192 (62)	100 (65)	92 (60)
Exclusive breast feeding	42	21 (14)	21 (14)
Age-appropriate immunization	213	108 (70)	105 (68)
Presence of smokers in house	198	97 (63)	101 (66)
Firewood used as cooking fuel	104	52 (34)	52 (34)
Nutritional edema	31	12 (8)	19 (12)
Severe stunting (Height for age Z score <-3SD)	68	39 (25)	29 (19)
Severe wasting (Weight for height Z score <-3SD)	43	20 (13)	23 (15)
Severe underweight (Weight for age Z score <- 3SD)	90	44 (29)	46 (30)
**Clinical findings during presentation**			
Fever	284	139 (90)	145 (94)
Diarrhea	276	137 (89)	139 (90)
Dehydration	90	41 (27)	49 (32)
Vomiting	58	32 (21)	26 (17)
Convulsion	40	20 (13)	20 (13)
Poor urination	58	31 (20)	27 (18)
Hypoxemia	48	25 (16)	23 (15)
Oral thrush	19	12 (8)	7 (5)
**Laboratory characteristics**			
Hemoglobin (Mean, ±SD)	289	10.68 ± 2.02	10.48 (± 1.98)
Total Leukocyte count, 10 × 10^4^ (median, * IQR)	289	12.92 (10.08, 17.58)	13.86 (10.78, 19.45)
Polymorph count (%) (Mean, ± ** SD)	289	48.55 ± 16.33	51.67 ± 15.46
Blood culture isolates	90 (17)	8/45 (18)	7/45 (16)
S. sodium (median, IQR) mmol/L	202	136 (132, 147)	137 (133, 149)
S. potassium (Mean, ±SD) mmol/L	202	3.96 ± 1.05	4.01 ± 1.24
TCO2 (Mean, ±SD)	202	15.41 ± 5.26	13.32 ± 5.63
S. creatinine (median, IQR) mmol/L	175	30.76 (23.32, 48.59)	29.70 (22.95, 61.20)

* Interquartile range; ** Standard deviation.

**Table 2:** Distribution of criteria of severe pneumonia, by treatment arms.

Variables	Overall (n = 308)	Amoxicillin Group (n = 154)	Ampicillin Group (n = 154)
Central cyanosis or hypoxemia (* SpO2 < 90%)	48 (16)	25 (16)	23 (15)
Severe Respiratory distress (Grunting or very severe chest indrawing)	92 (30)	41 (27)	51 (33)
General danger sign			
(i) Inability to breast-feed or drink	222(72)	116 (75)	106 (69)
(ii) Lethargy or unconscious, convulsion	94 (31)	41(27)	53 (34)

* peripheral capillary oxygen saturation.

**Table 3:** Comparison of the primary outcome, by treatment groups.

Variables	Overall	Amoxicillin Group	Ampicillin Group	RR	95% CI	*p*-Value
**Total treatment failure**	62 (20)	21 (14)	41 (27)	0.51	(0.32, 0.82)	0.004
(i) Persistence of ** danger signs (>48 h)	30 (10)	10 (6)	20 (14)			
(ii) Development of new ** danger signs (~24 h) (e.g.,*** Hypoxemia, severe sepsis or septic shock, or any general danger signs)	32 (11)	11 (7)	21 (14)			

** Danger signs are abnormal mentation, Inability to feeding, hypoxemia or cyanosis; *** Hypoxemia is defined by oxygen saturation < 90% in air.

**Table 4:** Reporting of un-expected outcome, by treatment arms.

Variables	Overall (n = 308)	Amoxicillin Group (n = 154)	Ampicillin Group (n = 154)	*p*-Value
Hospital outcome other than discharge	37	12 (8)	25 (16)	0.035
Referred	14	05 (3)	09 (6)	0.274
Left against medical advice (LAMA)	09	03 (2)	06 (4)	0.491
Death	14	4 (3)	10 (6)	0.101

**Table 5:** Comparison of secondary outcome (time to resolution of danger signs), by treatment arms.

Variables *	Amoxicillin Group (n = 154)	Ampicillin Group (n = 154)	*p*-Value
Time to resolution of hypoxemia(hours) (Median, IQR) (n = 60)	16.5 (8.0, 40.5)	19.0 (12.0, 45.5)	0.495
Fast breathing resolution in hours (Median, IQR) (n = 253)	16.0 (7.0, 36.0)	16.0 (7.0, 32.0)	0.688
General danger sign resolution in hours (Median, IQR) (n = 223)	41.0 (25.0, 49.5)	44.0 (24.0, 56.0)	0.489
Length of hospital stay (days) up to day 7	4.0 (3.00, 6.00)	4.00 (3.00, 6.00)	0.460

* Average hours and days.

**Table 6:** Survival analysis at day 7, by comparison between two treatment arms.

Variables	Overall (n = 308)	Amoxicillin Group	Ampicillin Group
Number of patients		154	154
Discharge from hospital		134 (87)	124 (81)
Censored *		20 (13)	30 (19)
Median duration of hospital stays at day 7 (IQR)	4.0 (3.0, 6.0)	4.0 (3.0, 6.0)	4.0 (3.0, 6.0)
Adjusted hazard ratio (95% CI)	1.05 (0.83, 1.35)		
*p*-value for hazard ratio	0.665		

* Death, refer outside, left against medical advice.

## Data Availability

The data set contained personal information of the study participants. Our institutional review board will not have the provision to disclose any kind of information. Thus, our policy is not to make availability of the data set in the manuscript, the supplemental files, or a public repository. However, data related to this manuscript are available upon request and for researchers who meet the criteria for access to confidential data may contact with Armana Ahmed to the research administration of icddr,b (http://www.icddrb.org/).

## Supplementary Materials

The following are available online at https://www.mdpi.com/article/10.3390/life11121299/s1, Supplementary Table S1: Reasons for exclusion of children with clinical signs of severe pneumonia.
