# Supplementary material for: Intravenous Amoxicillin Plus Intravenous Gentamicin for Children with Severe Pneumonia in Bangladesh: An Open-Label, Randomized, Non-Inferiority Controlled Trial
Source: Life (Basel). 2021 Nov 26;11(12):1299. doi: 10.3390/life11121299 (PMC8707665; doi:10.3390/life11121299)
Supplement: Supplementary file 1 [file life-11-01299-s001.zip › life-1420256-supplementary.pdf]

Supplementary Materials

Intravenous Amoxicillin Plus Intravenous Gentamicin for Children with Severe Pneumonia in Bangladesh: An Open-Label, Randomized, Non-Inferiority Controlled Trial

Lubaba Shahrin, et al.

Supplementary Table S1. Reasons for exclusion of children with clinical signs of severe pneumonia.

Reasons of exclusion	Total = 280<br>n (%)
Received antibiotics for >48 hours	198 (70.7)
Known congenital defects (including cardiac), obvious clinical features suggestive of chromosomal abnormality (Trisomy, microcephaly etc)	60 (21.4)
Terminally ill that required assisted ventilation or dialysis for AKI	28 (10)
Denial of giving informed consent by legal guardian	1 (0.3)
